# Utility of the Novel MediPost Mobile Posturography Device in the Assessment of Patients with a Unilateral Vestibular Disorder

**DOI:** 10.3390/s22062208

**Published:** 2022-03-12

**Authors:** Oskar Rosiak, Anna Gawronska, Magdalena Janc, Pawel Marciniak, Rafal Kotas, Ewa Zamyslowska-Szmytke, Magdalena Jozefowicz-Korczynska

**Affiliations:** 1 Balance Disorders Unit, Department of Otolaryngology, Medical University of Lodz, The Norbert Barlicki Memorial Teaching Hospital, 90-153 Lodz, Poland; anna.gawronska@umed.lodz.pl (A.G.); magdalena.jozefowicz-korczynska@umed.lodz.pl (M.J.-K.); 2 Audiology and Phoniatrics Clinic, Nofer Institute of Occupational Medicine, 91-348 Lodz, Poland; magdalena.janc@imp.lodz.pl (M.J.); ewa.zamyslowska@imp.edu.pl (E.Z.-S.); 3 Department of Microelectronics and Computer Science, Lodz University of Technology, 90-924 Lodz, Poland; pawel.marciniak@p.lodz.pl (P.M.); rafal.kotas@p.lodz.pl (R.K.)

**Keywords:** mobile posturography, vertigo, balance disorders, body-worn sensors, intraclass correlation coefficients, Bland–Altmann

## Abstract

Balance disorders are a growing problem worldwide. Thus, there is an increasing need to provide an inexpensive and feasible alternative to standard posturographic platforms (SP) used for the assessment of balance and to provide a possible solution for telemonitoring of patients. A novel mobile posturography (MP) MediPost device was developed to address these issues. This prospective study used a Modified Clinical Test of Sensory Interaction on Balance to evaluate healthy individuals and patients with a unilateral vestibular disorder through SP and MP simultaneously. The control group included 65 healthy volunteers, while the study group included 38 patients diagnosed with a unilateral vestibular deficit. The angular velocity values obtained from both methods were compared by intraclass correlation coefficients (ICC) and Bland–Altman plot analysis. Diagnostic capabilities were measured in terms of sensitivity and specificity. The ICC between the two methods for conditions 2–4 was indicative of excellent reliability, with the ICC > 0.9 (*p* < 0.001), except for Condition 1 (standing stance, eyes open) ICC = 0.685, *p* < 0.001, which is indicative of moderate reliability. ROC curve analysis of angular velocity for condition 4 represents the most accurate differentiating factor with AUC values of 0.939 for SP and 0.953 for MP. This condition also reported the highest sensitivity, specificity, PPV, and NPV values with 86.4%, 87.7%, 80%, and 90.5% for SP, and 92.1%, 84.6%, 77.8%, and 94.8% for MP, respectively. The newly developed MediPost device has high sensitivity and specificity in distinguishing between healthy individuals and patients with a unilateral vestibular deficit.

## 1. Introduction

Maintaining balance is a complex task that involves information from different peripheral receptors, which are then processed by the central nervous system. The information processed by cerebral and cerebellar regions responsible for balance includes vision, somatosensory input from the joints, and the vestibular system [1,2]. Recently, it has been noted that although equilibrium control is part of postural control, the two processes run parallel and are responsible for different phenomena. While “posture” refers to the distribution of tonic muscle activity, “equilibrium” compensates for perturbations, either internal or external [3]. Impairment in any of these systems might cause balance instability or vertigo and dizziness sensations. Because of this complexity, it is difficult to develop an instrument for measuring the efficiency of the balance system. 

Since several systems are integrated into the control of balance, there are many methods currently used for evaluating the dysfunction of balance. Since the discovery of the vestibulo-ocular reflex (VOR), first described by Hőgyes in 1881 [4], and further research into the physiology and pathophysiology of the vestibular organ by Bárány (for which he was awarded the Nobel Prize in 1914), one of the parameters primarily used in clinical practice nowadays is the disturbance of the VOR. Currently, visual integration into the balance system is mainly measured by means of videonystagmography (VNG). A complete VNG evaluation of the VOR comprises caloric and rotary chair testing. The examination also provides information about the integration of visual input into the vestibular system by tests of gaze stabilization and oculomotor function [5]. A thorough clinical evaluation of a dizzy patient also includes functional tests of vestibular mediated balance conducted by a physician, such as the Romberg test or the modified Clinical Test of Sensory Interaction and Balance (mCTSIB). The standing balance studies, a general term for such tests, are mostly based on the principle of subjecting the patient to an increasing difficulty in maintaining a standing posture such as removing visual information by closing the eyes (Romberg test) or somatosensory information by standing on foam (mCTSIB) [6,7]. If the patient can maintain standing posture in a defined time, the test is passed. With the development of sensor technology, measurements of the displacement of the center of gravity (body sway) in a two- or three-dimensional plane are now possible.

Currently, there is no golden standard for evaluating body sway [8]. One of the widely applied methods is posturography, which relies on tracking the displacement of subjects’ center of pressure (COP) projected onto a two-dimensional plane. Among the many output parameters in posturography, sway velocity, acceleration, sway path length, and area are most commonly analyzed [8,9]. Such an approach makes it possible to quantitively measure the patient’s ability to maintain a stable position under different conditions [10]

Over the years, posturography evolved from standard force-plate posturography (SP), which is used for evaluation in static conditions, to more complex devices such as computerized dynamic posturography (CDP) [11]. CDP enables researchers to evaluate body sway in dynamic conditions and to alter visual input; it provides additional information to conventional vestibular evaluation and can be used to diagnose a peripheral vestibular deficit [12]. The disadvantage of CDP is its high cost, which limits its clinical use to university centers.

Both SP and CDP provide an indirect measurement of the position of the center of mass (COM) because they are based on an inverted pendulum model; therefore, their output measurement is the COP [9,13]. Recently, the development of mobile technologies has allowed researchers to provide a more direct measurement of COM using mobile posturography (MP). There are several approaches to MP, but mostly these devices consist of an accelerometer and a gyroscope placed in the lumbar area [14]. MP allows for the evaluation of human balance in daily-life situations and more complex tasks [15], which is an important development towards monitoring patients with balance disorders or detecting falls or the exacerbation of symptoms via telemedical services.

Rehabilitation is of key importance to both central and peripheral vestibular disorders [16]. Both SP and CDP are efficient means of aiding the process. Clinical trials are underway to evaluate MP in the rehabilitation of patients with balance disorders [17]. Individually suited vestibular rehabilitation training decreases the sensation of vertigo, adapts the vestibulo-ocular reflex, and most importantly—improves stability and may reduce the risk of falls [18]. The latter is of utmost importance because falls contribute to increased mortality in the older adult population [19]. 

The American and British Geriatrics Society recommends screening for a history of falls in every person over 65 years of age and, in the case of individuals who report at least one fall over the last 12 months, an evaluation of gait and balance [20]. There are many solutions for non-instrumental tools for screening for risk of falls, such as the Timed up and Go test (TUG) or the Berg Balance Scale for the general assessment of balance. Such clinical tests provide a low-cost alternative to instrumental balance assessment; however, they remain highly subjective. Among the instrumental test for balance, recent studies focus on the mCTSIBprotocol for posturography and its application via mobile devices [21].

**Figure 1:**
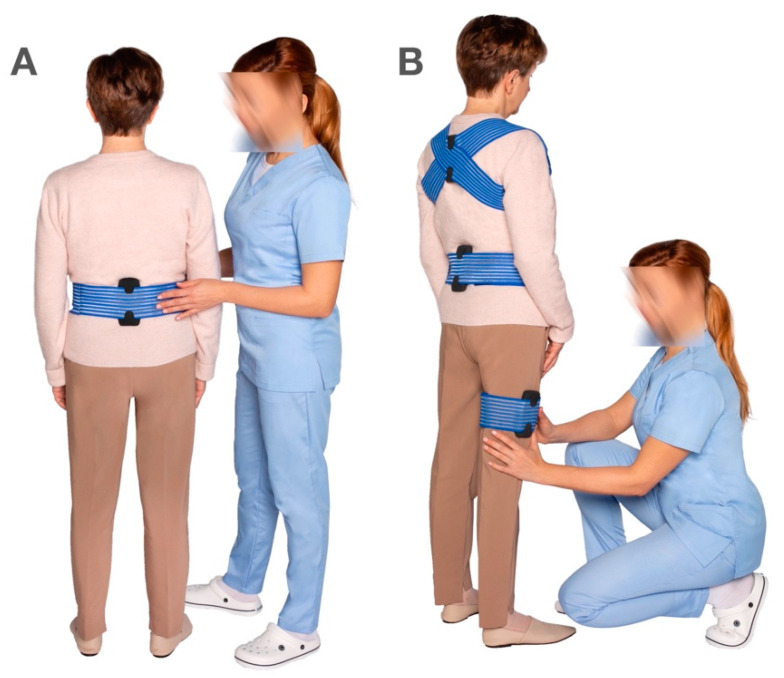
(**A**)—The MediPost device mounted in the lumbar area for single-sensor measurement, (**B**)—multiple MediPost sensors mounted on the trunk, lumbar area, and limbs for multi-sensor measurements.

The MediPost device was developed as a result of a multidisciplinary project “STRATEGMED” and funded by the National Center for Research and Development under the grant STRATEGMED2/266299/19/NCBR/2016 to develop a low-cost, mobile device capable of measuring human balance in dynamic conditions to facilitate the rehabilitation of patients with balance impairment. The device is designed to function either with one sensor mounted in the lumbar area (Figure 1A) or with several sensors mounted on the limbs, lumbar area, and the trunk to recreate a more complex biomechanical model for clinical studies (Figure 1B).

This study is a further expansion of the study conducted by our research group in 2019 [22] In our previous study, we established that MediPost can be an alternative to classical static posturography in terms of measurement reliability. This was achieved by analyzing compliance results of trajectories in specific tasks by means of intraclass correlation coefficients (ICC) and Bland–Altman plots. The ICC was 0.8 in the healthy control group and 0.9 in the group of patients with vestibular disorders.

**Figure 2:**
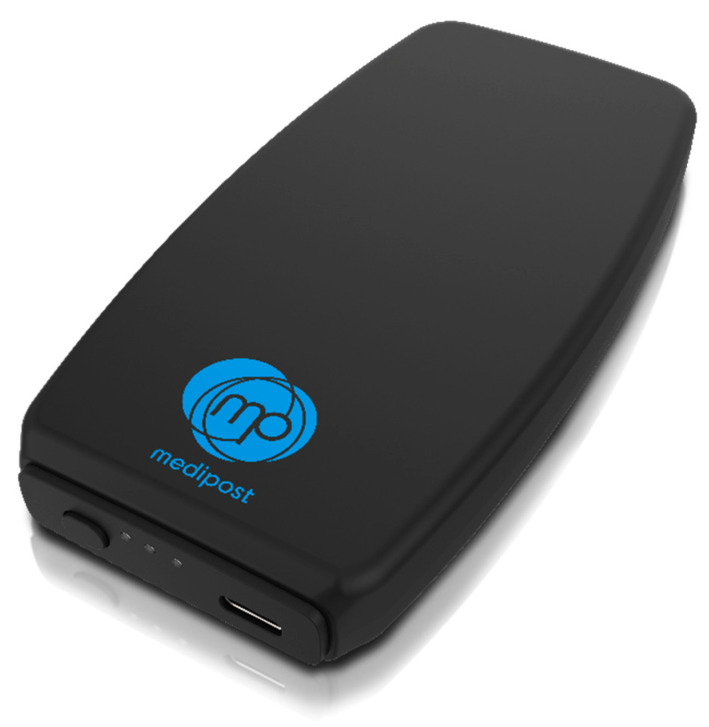
MediPost device, with a USB port for charging the device and battery status indicator on the shorter side of the device.

In this study, we expand on the compliance results between the two methods of measurement by analyzing each mCTSIB condition separately. We also focus on the MediPost’s ability to distinguish between healthy individuals and a homogenous group of patients with a unilateral vestibular deficit by reporting the sensitivity and specificity of each mCTSIB condition to detect a unilateral vestibular impairment. We decided not to include previously examined patients in this analysis because the device was updated with a new shell design in place of the prototype casing, now representing the final appearance (Figure 2). 

## 2. Materials and Methods

### 2.1. Ethics Approval

The research protocol was approved by the Bioethics Commission of the Medical University of Lodz (RNN/136/16/KE, 10.05.2016). All subjects provided written informed consent. All clinical investigation was conducted according to the principles expressed in the Declaration of Helsinki.

### 2.2. Sample Size Calculation

The sample size for this study was determined based on a preliminary experimental pilot study of 5 healthy volunteers and 5 patients after an episode of acute, unilateral vestibular deficit, where both groups were tested by SP and MP. The mean values from an SP standing on foam with eyes closed test (1.73 SD: 0.7) and the mean from the MP group (1.28) were then used to calculate the sample size, with the alpha of 0.05 and power of 90%. The determined sample size was 25 individuals with a recruitment ratio of 1.

Due to the ongoing nature of this project, more than the necessary 25 patients were included in this study. 

### 2.3. Control Group

The control group was recruited from healthy volunteers who signed informed consent to undergo clinical and VNG evaluations. The inclusion and exclusion criteria for the control group are summarized in Table 1.

The control group included 65 healthy volunteers, 52 females and 13 males, with a median age of 48 ±14 years. The average height was 166.64 ± 8.78 cm, the average weight was 73.94 ± 18.45 kg, and the average Body Mass Index (BMI) of 26.53 ± 6.05 bordered on obesity, with 36 individuals (55%) above the established normal BMI (25).

### 2.4. Study Group

Patients who were diagnosed with vertigo and balance instability at the Balance Disorders Unit, Otolaryngology Department, Medical University of Lodz, and who fulfilled the study requirements were subject to a prospective analysis. The study group included 38 patients, 19 females and 19 males, with a median age of 52 ± 22 years. 

The average height of the participants was 172.05 ± 9.64 cm, the average weight was 77.16 ± 18.13 kg, and the average Body Mass Index (BMI) of 25.88 ± 4.98 bordered on obesity, with 20 individuals (52%) above the established normal BMI (25).

Patients with peripheral vestibular dysfunction present in clinical examination, with symptoms lasting no more than 4 weeks, were included in the study group. Vestibular dysfunction was confirmed in videonystagmography (VNG) under bithermal (44° and 30 °C) water caloric test (VNG Ulmer, Synapsis, Marseille, France). Canal paresis (CP) asymmetry greater than 30% was diagnostic as a peripheral vestibular deficit. 

The saccades, smooth pursuit, and optokinetic non-caloric tests were performed to assess if there were any abnormalities suggestive of a central vestibular disorder. The inclusion and exclusion criteria for both study groups are summarized in Table 1. 

### 2.5. Posturography Evaluation

The mCTSiB protocol [7] was used in this study, which was performed on the NeuroCom^®^ Static Balance Master posturography (NeuroCom^®^ International Inc., Clackmas, OR, USA) (SP) equipment. The mCTSIB was recorded simultaneously with a MediPost mobile posturography device (MP) attached at the lumbar level. 

**Figure 3:**
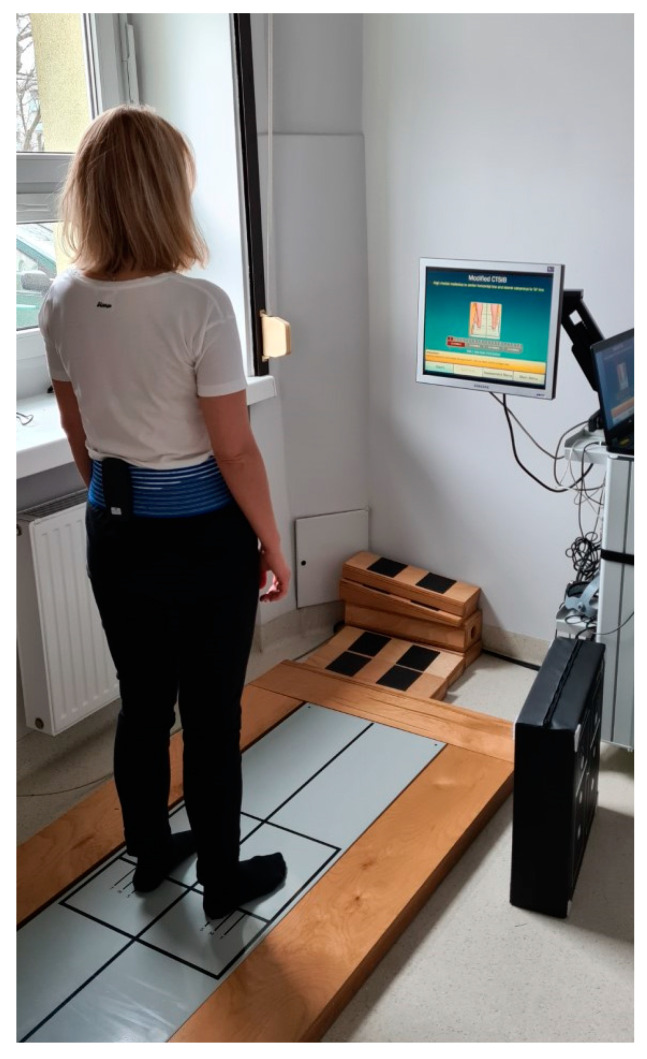
The study setup. The subject is standing in quiet stance on a static posturography plate, feet apart, with the MediPost device mounted in the lumbar area. Visual instructions are presented on the screen in front of the patient.

The subjects were asked to stand with their hands at their sides, feet apart, and to perform the following 4 sensory conditions: standing on a firm (Condition 1) and foam (Condition 3) surface with eyes open; standing on a firm (Condition 2) and foam (Condition 4) surface with eyes closed. Every condition included 3 repetitions lasting 10 s; a mean value was then calculated. If the subject was not able to maintain balance during the test, their fall was automatically marked by the software, and the value of 6°/s was substituted in the result sheet. The same principle was applied to MediPost. A composite score was calculated as a mean from all mCTSIB conditions for each subject. Both measurements were conducted simultaneously. The setup is presented in Figure 3.

### 2.6. MediPost

The MediPost is a portable, battery-powered device controlled by an ESP32 microprocessor system with a Wi-Fi radio module. It uses a 3-axis IMU (Inertial Measurement Unit) to determine orientation in space. The IMU (STMicroelectronics LSM9DS1) contains a microelectromechanical system (MEMS), consisting of an accelerometer, gyroscope, and magnetometer. The device is connected wirelessly (Wi-Fi) to a computer and is synchronized and controlled by a software application. The application connects to a predefined Wi-Fi network via a defined port. The sampling rate used in the IMU is 200 Hz. A low-pass filter has been implemented in the IMU to remove noise. Finally, the ESP32 system sends a signal represented by 20 samples per second to the computer. Samples containing information from the three axes are sent when the measurement is completed. The Madgwick IMU algorithm [23] was chosen to determine the angular position of the device. This algorithm is based on the use of quaternions to represent orientation in 3D space. A detailed description of the system can be found in [22]

### 2.7. Data Analysis and Statistical Methodology

Data were stored on a computer and analyzed using Statistica 13.1 Software (TIBCO Software Inc, Palo Alto, CA, USA). The angular sway velocity [°/s] was the main outcome both from platform posturography and MediPost, as reported in other studies on this subject [15]. Continuous variables were tested for normality of distribution using the Shapiro-Wilk test. An alpha level of 0.05 was established. For non-normally-distributed variables, the Wilcoxon test was used for comparing paired samples, and the U-Mann-Whitney test for non-paired comparisons. The median and interquartile range (IQR) was analyzed, and the values were power transformed using Box-Cox methodology for evaluating the intra-class correlation coefficient (ICC). The ICC was measured using a two-way mixed-effects absolute-agreement average-measurement model and interpreted using criteria proposed by Koo et al., where values less than 0.5, between 0.5 and 0.75, between 0.75 and 0.9, and greater than 0.9 are indicative of poor, moderate, good, and excellent reliability, respectively [24]. To assess the clinical utility for both methods, ROC curves were analyzed, and the Youden index was used to establish cut-off values for continuous variables. The Area Under Curve (AUC) and AUC difference were used to compare ROC curves for both methods; an algorithm by Hanley [25] was used to test for statistical significance of the differences between ROC curves for each mCTSIB condition. The criterion of statistical significance *p* < 0.05 was used in the statistical analyses. 95% Confidence Interval values were provided where applicable.

## 3. Results

To compare SP and MP, the angular velocity median values measured by NeuroCom SP and MediPost were analyzed. Comparing the median values between all four conditions under SP, without distinguishing the control group and the study group, we observed that the angular velocities provided employing SP and MP were different (*p* < 0.001). The difference between both methods was most visible under Condition 4, where the median angular velocities differed by 0.24°/s, and least visible under Condition 3, where median values were the same (0.55°/s). However, the interquartile range was narrower in the MP mobile posturography measurements. When comparing the angular velocity values acquired for the study and control groups with the same measurement method, we observed higher average velocities in the group with vestibular impairment, with the exception of Condition 1, where no differences were observed. 

The ICC among the two methods for mCTSIB test conditions 2-4 and the composite score were indicative of excellent intraclass reliability with the ICC > 0.9 (*p* < 0.001), except for Condition 1 (standing stance, eyes open) ICC = 0.685, *p* < 0.001, which is indicative of moderate reliability. The mean difference between the MP measurement and SP measurement and the standard deviation of the difference is graphically presented on Bland–Altman plots for each of the four mCTSIB test conditions. Median values of angular velocities and corresponding ICC are presented in Table 2.

**Figure 4:**
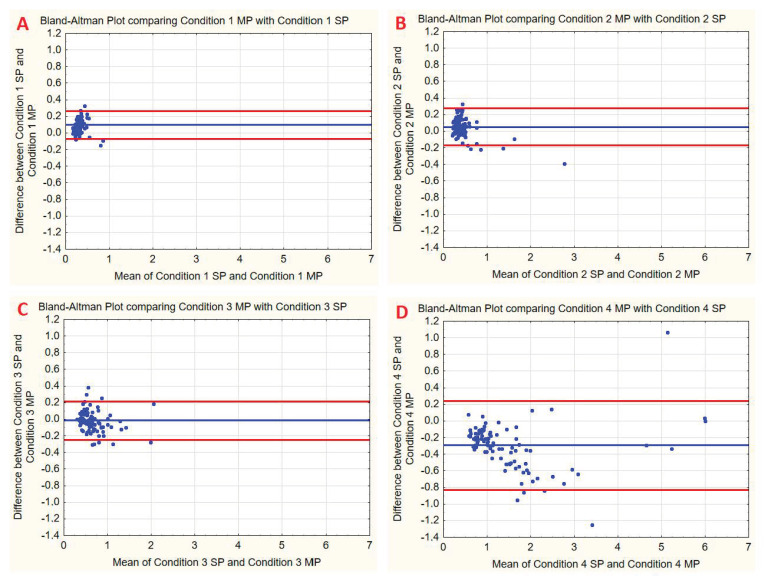
Bland–Altman plots for the mean difference of angular velocity measurement using two methods: mobile posturography with the MediPost and static posturography. Blue lines represent bias, red lines represent limits of agreement. (**A**) mCTSIB test condition 1. (**B**) mCTSIB test condition 2. (**C**) mCTSIB test condition 3. (**D**) mCTSIB test condition 4. SP- Static Posturography. MP-Mobile Posturography with MediPost.

To check the agreement between SP based on tensometers and MP, based on inertial sensors, the Bland–Altman plot and coefficient were also used. The values obtained using this method are presented in Table 3, the Bland-Altman plots are presented in Figure 4. It should be noted that the literature does not always present a uniform approach regarding threshold values required to pronounce satisfactory agreement. In this respect, interpretation of the Bland–Altman coefficient usually follows the original study [26], in which 95% is given as the threshold. Considering this, the authors assumed that results at 95% and slightly below could be considered satisfactory. 

The Bland–Altman plot shows agreement between static SP and MP for four conditions. The blue line indicates the mean difference between these two methods. The red lines represent differences expressed in terms of standard deviation (SD): +1.96 SD and −1.96 SD.

To establish the clinical utility of MediPost, we analyzed the diagnostic performance of this device by ROC curve analysis. We also compared ROC values for SP and MP in a two-way test to determine which of the two methods performs better in distinguishing a unilateral vestibular disorder. The results of the ROC curve analysis for all posturography conditions are presented below in Table 4. 

Among all mCTSIB conditions in the ROC curve analysis, condition 4 represents the most accurate differentiating factor, with AUC values of 0.939 for static posturography and 0.953 for MediPost. This condition also reported the highest sensitivity, specificity, PPV, and NPV values, with 86.4%, 87.7%, 80%, 90.5% for SP, and 92.1%, 84.6%, 77.8%, 94.8% for MediPost, respectively. The difference between AUC values for both methods was 0.014 in favor of MediPost; this difference was not considered statistically significant (*p* = 0.162). 

Condition 1 presented the worst diagnostic performance with the smallest AUC values of 0.679 for SP and 0.573 for MediPost; the difference between AUC values for both methods was 0.106. Although the AUC value for SP was higher, it was not statistically significant (*p* = 0.067). The composite score may serve as a reliable diagnostic parameter (ICC 0.981; *p* < 0.001, AUC 0.940 for SP and 0.954 for MediPost). Under all mCTSIB conditions, there were no statistically significant differences between the AUC values for the ROC curves, which implies that the methods are clinically equivalent. 

## 4. Discussion

In line with the results of our previous study from 2019, we have again confirmed the agreement between static and mobile posturography. In a similar study, Valldeperes et al. [27] evaluated an MP device where the overall intra-class correlation index was 0.93. This is similar to our study, with an ICC of 0.96, which is indicative of excellent reliability, except for Condition 1 (firm stance, eyes open) with a lower ICC of 0.68. An important aspect raised by Valldeperes et al. was whether the filter applied to compensate for sensor drift could be applied to individuals with a balance dysfunction due to different sway frequencies than those of a normal subject. In our study, the device also uses a Kalman filter and was still able to provide a good evaluation of patients with impaired balance.

This study determined that the angular velocity was significantly higher in patients with a unilateral vestibular dysfunction in mCTSIB Conditions 2–4. Similar findings have been reported by Varela et al. [28] in a study using a tridimensional electromagnetic sensor mounted at the sacral region, where a group of patients with vestibular impairment demonstrated increased trunk sway velocity when compared to a healthy control group. The average age and BMI values were similar to the group in our study. Although the devices were not mounted in exactly the same area, the results are concordant to those obtained in this study, which could imply that a single MP sensor can be mounted either in the lumbar or sacral region. In our observation, placement in the lumbar region allows for better fixation of the sensor with the band, thus preventing distortion in measurements caused by sensor drift.

Another study by Baloh et al. [29] determined increased trunk sway velocities in a clinical test in quiet stance with eyes closed on a foam surface in a group of older adults with vestibular disfunction when compared to age-matched controls. It was noted, however, that trunk sway velocities were also significantly higher in older adults (mean age 80.8) than in a group of healthy young adults (mean age 26.6). Thus, sway velocity cannot be used as a sole screening test in individuals who do not report balance issues.

In this study, we determined cut-off values for healthy individuals and patients with a unilateral vestibular deficit. Moreover, we have established the clinical performance of both methods through sensitivity, specificity, and ROC analysis. Depending on the particular test condition in the mCTSIB protocol, the performance values were clinically unsatisfactory (condition 1) or highly sensitive and specific (Condition 4). The composite score from the entire mCTSIB test yields optimal results with similar sensitivity and specificity (89.5% and 84.6% for SP; 87.7% for MP). These findings are in line with other studies on the mCTSIB protocol, where Condition 4 (or an equivalent test in quiet stance with eyes closed on a foam surface) showed the best diagnostic performance for vestibular dysfunction [30]. 

In a review on measuring vestibular contributions to balance impairment by Wagner et al. [31], it was noted that digitalized posturography tools improve the sensitivity of clinical balance tests in quiet stance. The examination could be limited to only performing mCTSIB Condition 4, as it yields similar diagnostic performance to a composite score and improved performance over Conditions 1–3. An automated approach to such measurement performed in an outpatient setting or a general practitioners office could provide quantitive information in follow-up examinations of vestibular patients [32] with the possibility of observing an improvement after home-administered rehabilitation or deterioration in vestibular function requiring a referral to a specialist.

Mobile posturography can be a promising solution to the growing problem of an aging society with balance disorders. Most vestibular and balance dysfunction rehabilitation programs can be performed at home after initial training with a physiotherapist, the results of such exercises could be monitored remotely. Furthermore, the advantage of wireless, multi-sensor mobile posturography over force-plate systems is the possibility of tracking movement and gait disturbances. Such capabilities could provide additional information in research on gait abnormalities in patients with Parkinson’s disease [33,34,35] or center of mass data in sports medicine [36]. As noted in a review of state-of-the art balance sensors by Ma et al. [37] future trends in development of such devices should focus on making them more lightweight and with higher computing power. The MediPost is a valuable improvement in research on mobile posturography.

## 5. Conclusions

The newly developed MediPost mobile posturography device has high sensitivity and specificity in distinguishing between healthy individuals and unilateral vestibular deficit by conducting the mCTSIB protocol. Condition 4 and its composite score provide the best diagnostic results for distinguishing between healthy and balance-impaired individuals. 

Future research should include patients with other vestibular pathologies, possibly with the use of the multiple MediPost sensor setup to study the possible relationship between COM and lower-limb movement. The cable-free design of the device allows the clinician to study everyday activity or monitor posture data during sports activity or rehabilitation exercises, which is unavailable for either static or dynamic posturography.

### Study Limitations

The vestibular dysfunction group recruited for this study involved only unilateral vestibular deficit adult patients. Due to the limitations of static posturography, this research included only tasks in quiet stance. Further research should include more numerous populations of older adults with age-matched controls. 

## Figures and Tables

**Table 1:** Inclusion and exclusion criteria for recruitment to study and control groups.

	Control Group (Healthy Volunteers)	Study Group (Vestibular Disorder)
Inclusion criteria	CP < 30% in VNG Ability to perform posturographic evaluations	CP ≥ 0% in VNG Acute vertigo and balance problems of at least 2 weeks duration Persistent feeling of instability
Exclusion criteria	Any abnormalities present in VNG Known neurological or other serious diseases, e.g., circulatory and musculoskeletal system	Presence of central vestibular abnormalities in oculomotor VNG tests in more than 2 tests. Musculoskeletal or orthopedic pathology interfering with proper posturography evaluation An acute or chronic hearing disorder

CP—canal paresis; VNG—videonystagmography.

**Table 2:** Median values of angular velocity under four posturography conditions measured by mobile posturography (MediPost) and static posturography.

Posturography Condition	Static Posturography Median (IQR) [°/s]			Mobile Posturography MediPost Median (IQR) [°/s]			*p* Value Wilcoxon Test	Intra-class Correlation Coefficient (ICC) [95%CI]
**Group**	All patients (n = 103)	Control (n = 65)	Study (n = 38)	All patients (n = 103)	Control (n = 65)	Study (n = 38)		
**Condition 1**	0.23 (0.07)	0.23 (0.03)	0.26 ^ns^ (0.13)	0.35 (0.13)	0.35 (0.13)	0.36 ^ns^ (0.17)	<0.001	0.685 * (0.087; 0.875)
**Condition 2**	0.33 (0.13)	0.27 (0.13)	0.43 * (0.17)	0.42 (0.14)	0.37 (0.11)	0.48 * (0.15)	<0.001	0.958 * (0.922; 0.976)
**Condition 3**	0.55 (0.3)	0.47 (0.17)	0.78 * (0.36)	0.55 (0.23)	0.49 (0.15)	0.73 * (0.39)	<0.001	0.958 * (0.939; 0.972)
**Condition 4**	1.17 (1.0)	0.97 (0.30)	2.15 * (1.10)	0.93 (0.67)	0.79 (0.29)	1.61 * (1.02)	0.049	0.966 * (0.702; 0.989)
**Comp**	0.6 (0.4)	0.50 (0.20)	0.90 * (0.30)	0.54 (0.29)	0.49 (0.92)	0.84 * (0.32)	<0.001	0.981 * (0.964; 0.989)

Comp—composite score, mean value for 4 conditions, *—significant at the <0.001 level (2-tailed), ^ns^- no statistical significance.

**Table 3:** Compliance results between MediPost and static posturography.

Modified Clinical Test of Sensory Interaction on Balance Condition	Bland–Altman Coefficient [%]
1	94.17
2	95.14
3	92.23
4	95.14

**Table 4:** Median values of angular velocity under four posturography conditions measured by mobile posturography (MediPost) and static posturography.

Modified Clinical Test of Sensory Interaction on Balance		AUC (95% CI)	Proposed Cut-Off Value	AUC Difference	Comparison of ROC Curves (*p* Value)	Sensitivity	Specificity	PPV	NPV
**Condition 1**	SP	0.679 (0.566; 0.793)	0.27	0.106	0.067	36.8%	89.2%	66.7%	70.7%
	MP	0.573 (0.452; 0.692)	0.45			26.3%	93.8%	71.4%	68.5%
**Condition 2**	SP	0.813 (0.728; 0.899)	0.4	0.039	0.391	71.1%	78.5%	65.9%	82.3%
	MP	0.773 (0.675; 0.872)	0.43			76.3%	76.9%	65.9%	84.7%
**Condition 3**	SP	0.905 (0.848; 0.961)	0.7	0.035	0.170	71.1%	93.8%	87.1%	84.7%
	MP	0.870 (0.802; 0.937)	0.58			76.3%	78.5%	67.4%	85%
**Condition 4**	SP	0.939 (0.896; 0.982)	1.53	−0.014	0.166	86.4%	87.7%	80%	90.5%
	MP	0.953 (0.918; 0.989)	1.08			92.1%	84.6%	77.8%	94.8%
**Composite score**	SP	0.94 (0.897; 0.983)	0.7	−0.014	0.365	89.5%	84.6%	77.3%	93.2%
	MP	0.954 (0.92; 0.989)	0.61			89.5%	87.7%	81%	93.4%

AUC—area under curve, PPV—positive predictive value, NPV—negative predictive value, SP—Static posturography, MP—Mobile posturography (MediPost device).

## Data Availability

The data presented in this study are available on request from the corresponding author. The data are not publicly available due to restrictions imposed by the funding institution.
